# Supplementary material for: Plekhg5-regulated autophagy of synaptic vesicles reveals a pathogenic mechanism in motoneuron disease
Source: Nat Commun. 2017 Oct 30;8:678. doi: 10.1038/s41467-017-00689-z (PMC5662736; doi:10.1038/s41467-017-00689-z)
Supplement: Supplementary file 1 — Supplementary Information [file 41467_2017_689_MOESM1_ESM.pdf]

Description of Supplementary Files

File Name: Supplementary Information
Description: Supplementary Figures.

File Name: Supplementary Movie 1
Description: Autophagosome-movement in wild-type motoneurons.

File Name: Supplementary Movie 2
Description: Autophagosome-movement in Plekhg5-deficient motoneurons. For monitoring the axonal transport of autophagosomes, motoneurons were transduced with GFPRFP-LC3 and cultured for seven days. At day seven the movement of GFP-LC3 structures along axons was monitored for 20 min.

File Name: Peer Review File

Supplementary Figure 1

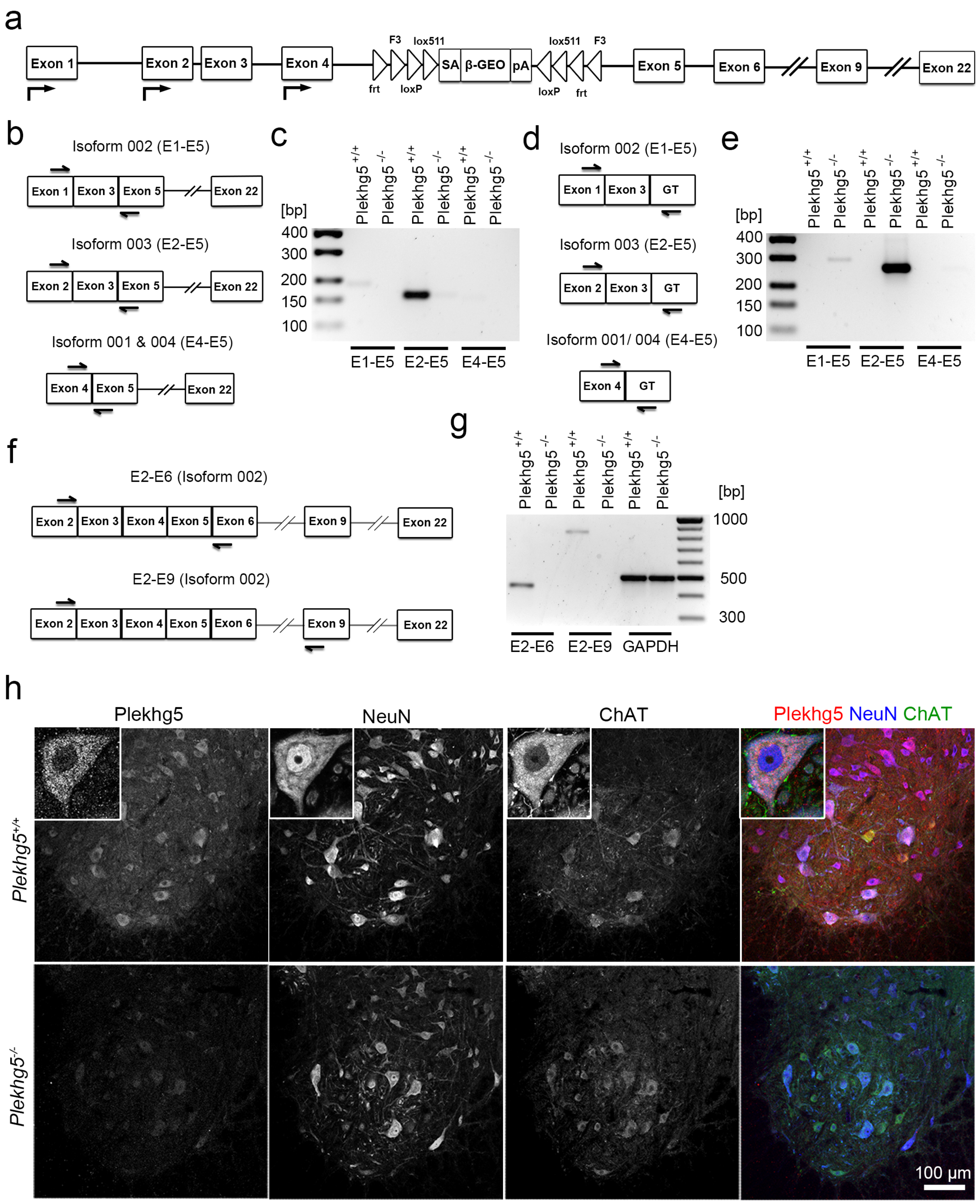

Generation of Plekhg5 deficient mice. (**a**) Scheme of the mutant *Plekhg5* locus. The gene trap cassette (GT-cassette) consisted of a splice acceptor, a *β-geo* gene and a poly-adenylation signal. Correct insertion of the GT-cassette within the *Plekhg5* locus was verified by sequencing. According to ensemble.org, the murine *Plekhg5* locus is able to generate four different protein-coding transcripts (isoforms 001-004) with three distinct N-termini. Isoform

001 and 004 only differ in their PDZ-binding motif, comprising three amino acids at the C-terminus. The GT-cassette is inserted within the fourth intron of the *Plekhg5* gene and affects transcription of all predicted splice variants. Frt/F3 flippase recognition target; loxP/lox511 Cre recognition sites; SA, splice acceptor; pA, bovine growth hormone polyadenylation sequence; β-GEO, β-galactosidase/neomycin phosphotransferase fusion gene. (**b - d**) Splice events at the wildtype (**b, c**) and gene-trap (**d, e**) locus, characterized by RT-PCR. To investigate which isoforms are expressed within the spinal cord, RT-PCRs were carried out with specific 5´ primers for each splice variant. For the wildtype-allele one common 3´ primer was used, located in the next downstream exon (ENSMUSE00000594961) of the GT-cassette (**b**). For detection of the GT-cassette a specific 3´ primer was applied (**d**). In wildtype-animals, *Plekhg5* is dominantly expressed within the spinal cord as isoform 003 (E2-E5) (**c**). We hardly detected any *Plekhg5* transcripts in homozygous mutant animals (**c**). Conversely, expression of the GT-cassette was only detectable in mutant animals (**e**). Thus, the gene-trap locus predominantly generates a truncated protein consisting of 28 amino acids (Exon 2 and 3 of isoform-003) fused to β-GEO. The 28 amino acids of the original protein contain neither the RhoGEF nor PH-domain. (**f**, **g**) Using 5´ primers specific for exon 6 and exon 9 (**f**, **g**) we confirmed that insertion of the GT-cassette did not disturb the normal splicing pattern by altering the order of 5´ exons or elimination of 5´ exons (**f**, **g**). These data demonstrate functionality of the GT-cassette and confirm the absence of wildtype-transcripts within the spinal cord of mutant mice. (**h**) Using an antibody directed against the C-terminus of Plekhg5, we detected a vesicular staining within the cytosol. As indicated by co-staining against NeuN and choline acetyltransferase (ChAT), expression of Plekhg5 was identified in $NeuN^+$ neurons including $ChAT^+$ motoneurons. In *Plekhg5*$^{-/-}$ mice, Plekhg5 staining was completely abolished, providing further evidence for functionality of the GT-cassette.

Supplementary Figure 2

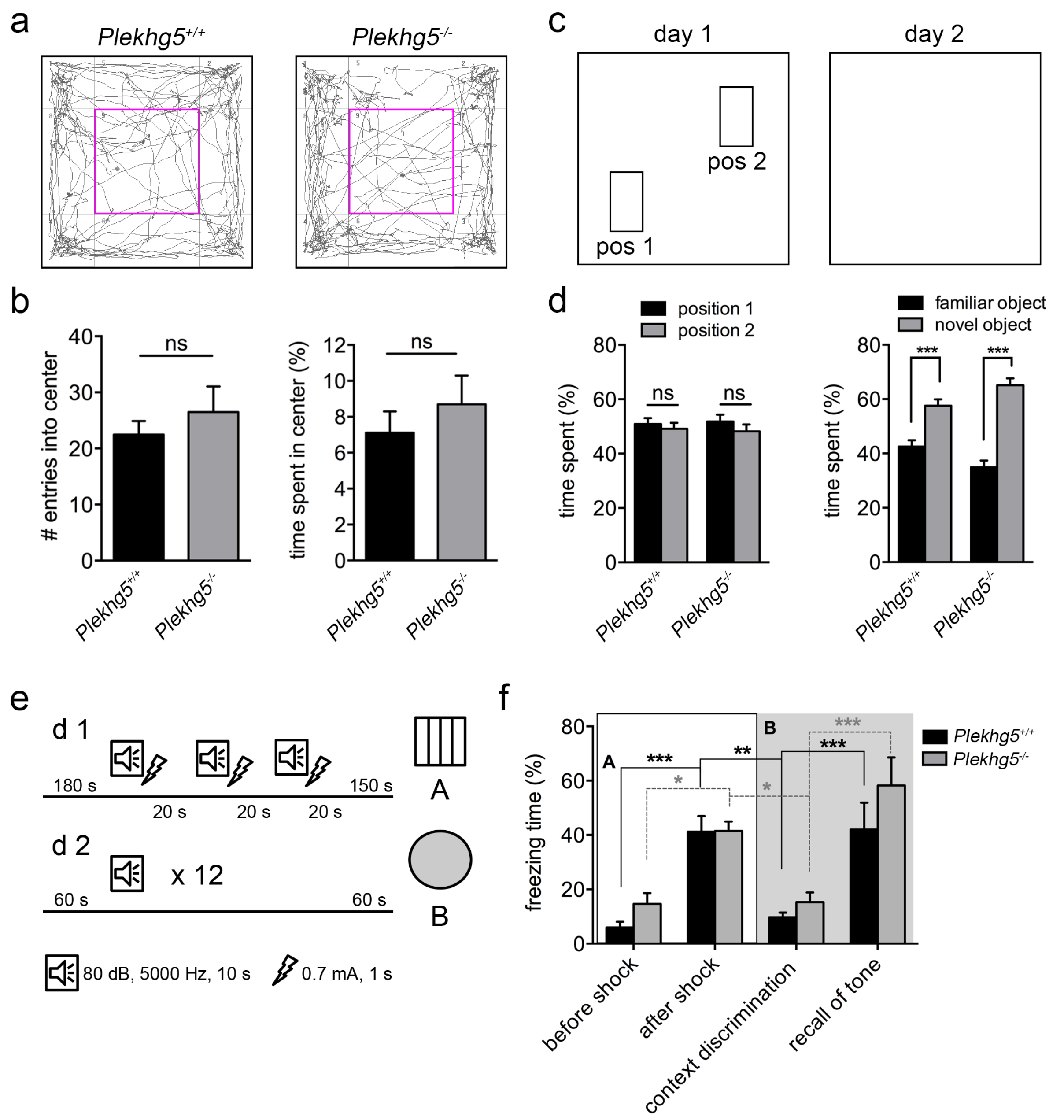

Plekhg5 deficiency does not impair cognitive function. (**a**, **b**) Control- and Plekhg5 deficient mice were placed in the center of the open field arena and their movement was monitored for 10 minutes. (**a**) Representative trajectories of control- and Plekhg5 deficient mice show the behavior of both groups in the open field test. (**b**) Plekhg5 deficient mice spent the same amount of time in the center as compared to control animals. Both groups also showed the same number of entries into the center. (**c**, **d**) To assess the memory skills of Plekhg5 deficient mice the object recognition test was performed. (**c**) The first day two identical objects were presented and placed in two diagonally opposing corners of the box. Mice were

placed in the middle of the box and monitored and tracked for 10 minutes. The next day one of the objects was replaced with a novel object. Whether the novel object is presented at position 1 or 2 was randomized to avoid position bias. Again, mice were placed in the middle of the box and monitored and tracked for 10 minutes. (**d**) At day 1, control- and Plekhg5 deficient mice spent half of the time at both objects. The second day both groups spent significantly more time at the novel object suggesting that both groups are able to recall the familiar object and recognize the new object. (**e**, **f**) We investigated fear acquisition and context discrimination of Plekhg5 deficient mice in a paradigm of fear conditioning. (**e**) At day 1, mice were trained to associate a tone with a foot shock in context A. After a 180 s habituation phase, mice received a 10 s tone of which the last second is accompanied by an electric foot shock delivered via the metal grid in the cage. The stimuli are presented 3 times with an inter stimulus interval (ISI) of 20 s. At day 2, mice have to recall the acquisition in context B. After a 60 s habituation phase, mice receive a 10s tone that is not accompanied by an electric foot shock. The sound is presented 12 times with an ISI of 20s. (**f**) Quantification of freezing time. During acquisition (context A) both groups show a significant increase in freezing time after receiving an electric foot shock. At day 2 both, control and Plekhg5 deficient mice are able to recall the acquisition in context B. Both groups show a significantly increase in freezing time after receiving the tone without electric foot shock.

Supplementary Figure 3

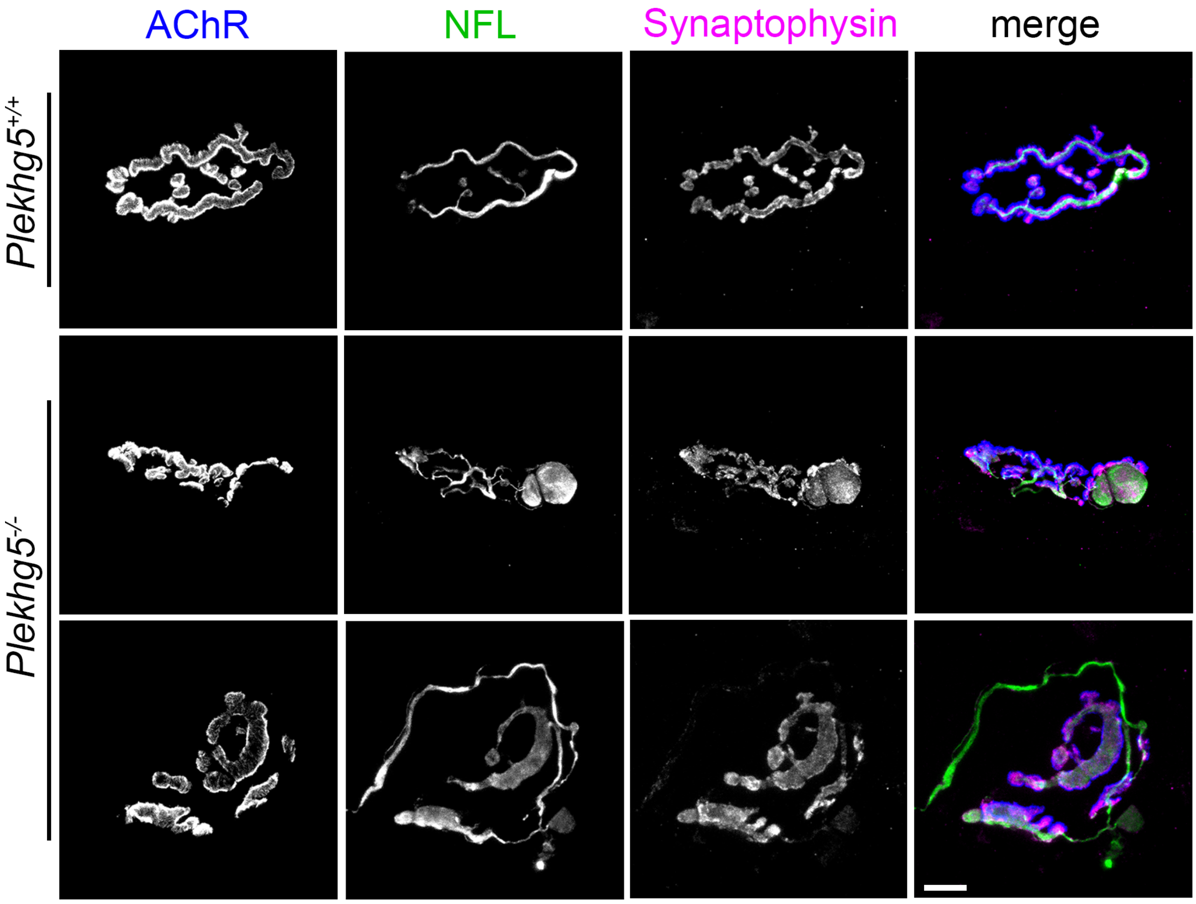

Degradation of neuromuscular junctions in Plekhg5 deficient mice. Representative images of neuromuscular junctions within the gastrocnemius muscle of adult mice stained for acetylcholine receptors (BTX labeling), NFM and synaptophysin. Scale bar: 10 µm.

Supplementary Figure 4

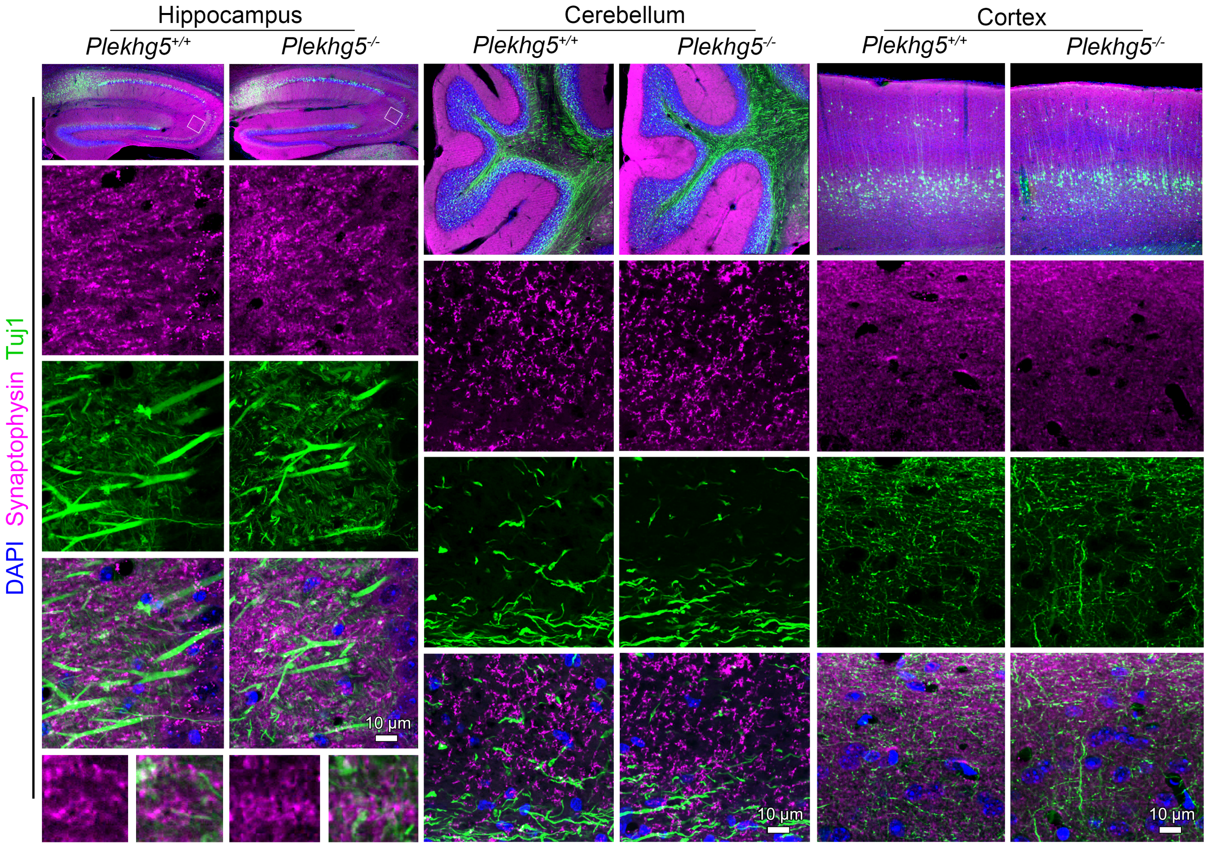

Histological analysis of major brain areas. Brain sections of control and Plekhg5 deficient mice were stained for synaptophysin, Tuj1 and DAPI. The morphology and layering of major brain areas as well as the synapse morphology appeared normal in Plekhg5 deficient mice as compared to controls animals.

Supplementary Figure 5

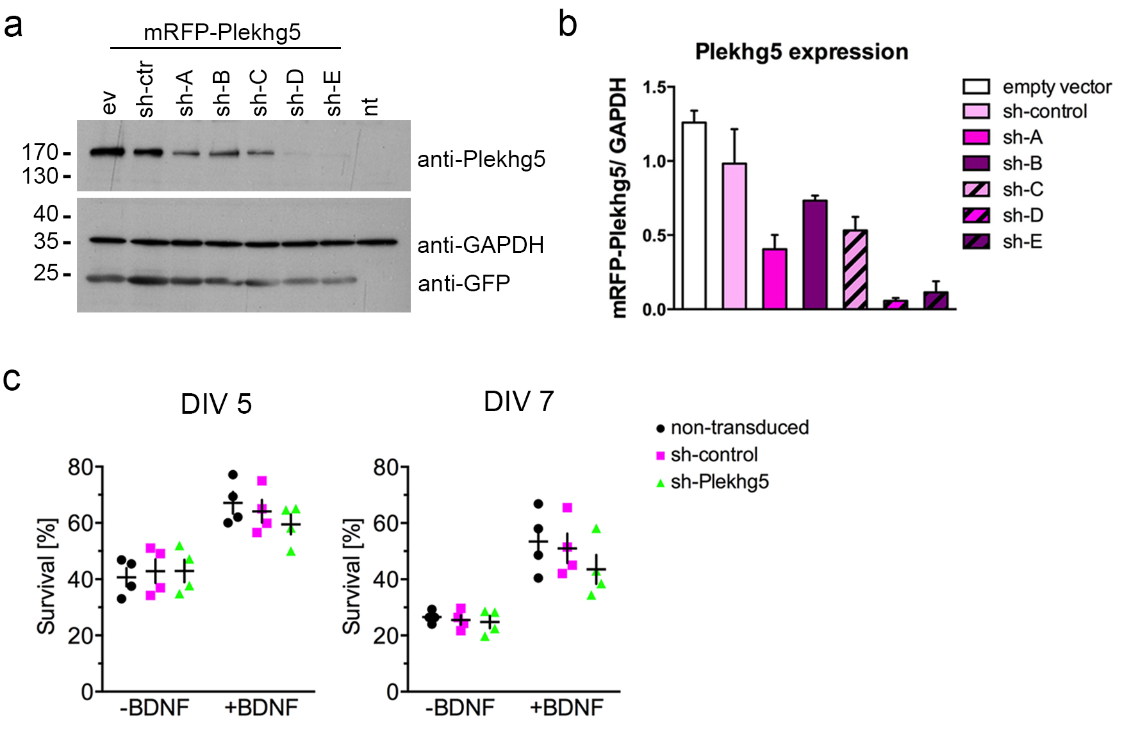

Depletion of Plekhg5 does not impair survival of cultured motoneurons. (**a**, **b**) Different sh-RNA constructs were validated for their ability to knockdown Plekhg5. Co-transfection of HEK293T cells with mRFP-Plekhg5 and different sh-RNAs targeting Plekhg5. After 48h, cells were lysed and submitted to SDS-PAGE and Western blotting. Images have been cropped for presentation. Full size images are presented in Supplementary Fig. 7. (**c**) After five and seven days, knockdown of Plekhg5 with construct # D did not significantly affect motoneuron survival when cultured with or without BDNF (each data point represents one individual experiment; Mean ± SEM; Two-way ANOVA; Bonferroni post-test).

Supplementary Figure 6

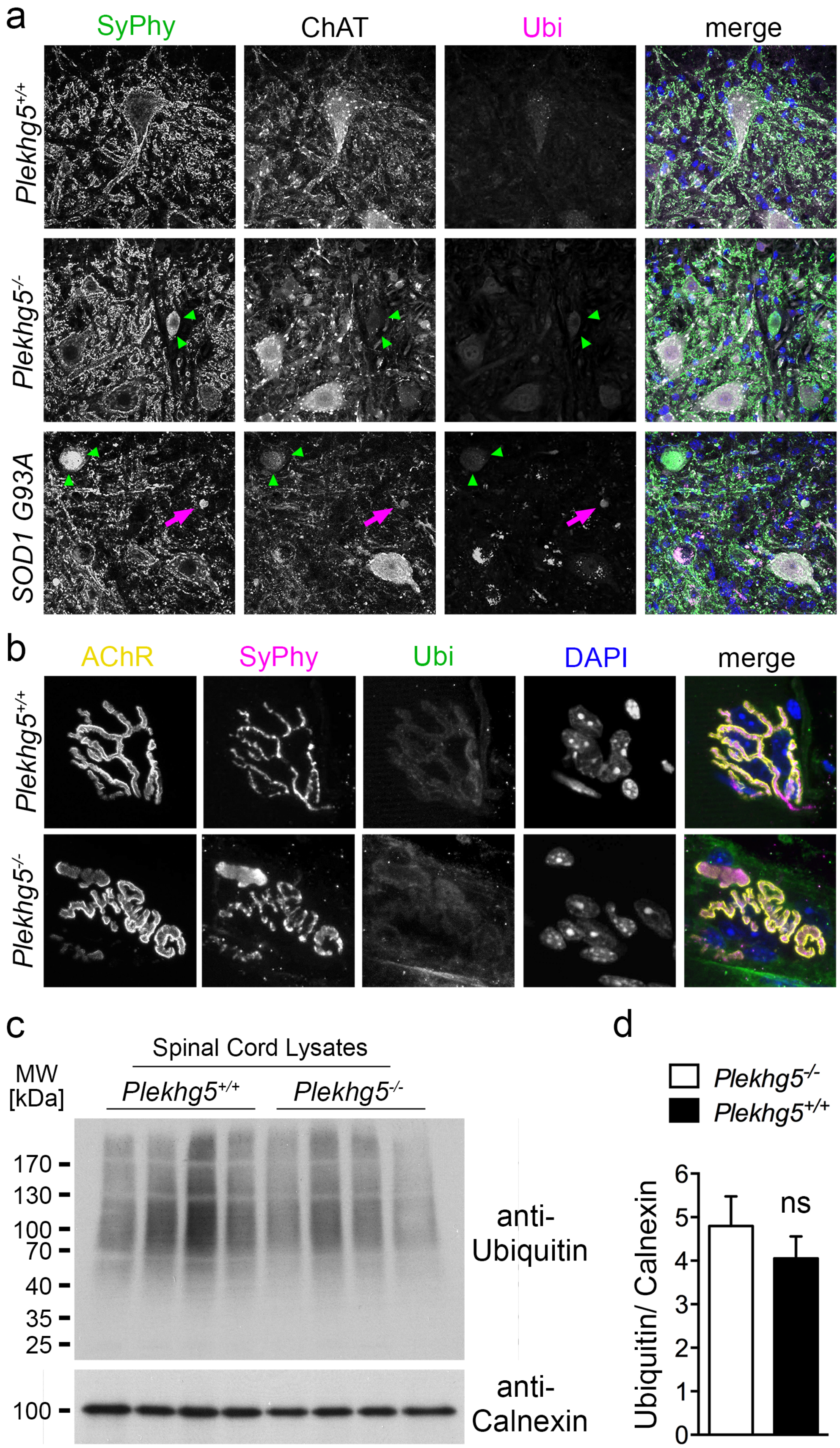

Global protein homeostasis is not impaired by Plekhg5 deficiency. (**a**) Ubiquitin staining of spinal cord cross-sections from control-, Plekhg5 deficient- and SOD1 G93A mice. Plekhg5 deficient mice showed no enrichment for polyubiquitinated protein inclusions, in contrast to SOD1 G93A mice. Arrowheads point to Synaptophysin accumulations. Arrows point to accumulations positive for Synaptophysin and Ubiquitin. (**b**) Ubiquitin staining of NMJs. Ubiquitinated protein inclusions were not detectable at NMJs of Plekhg5 deficient mice. (**c**) Western bot analysis of spinal cord extracts probed for Ubiquitin. Plekhg5 deficient mice did not reveal any enrichment for polyubiquitinated proteins. (**d**) Western blot quantification. (Four animals per genotype were analyzed; Mean ± SEM; unpaired t-test; two-tailed)

Supplementary Figure 7

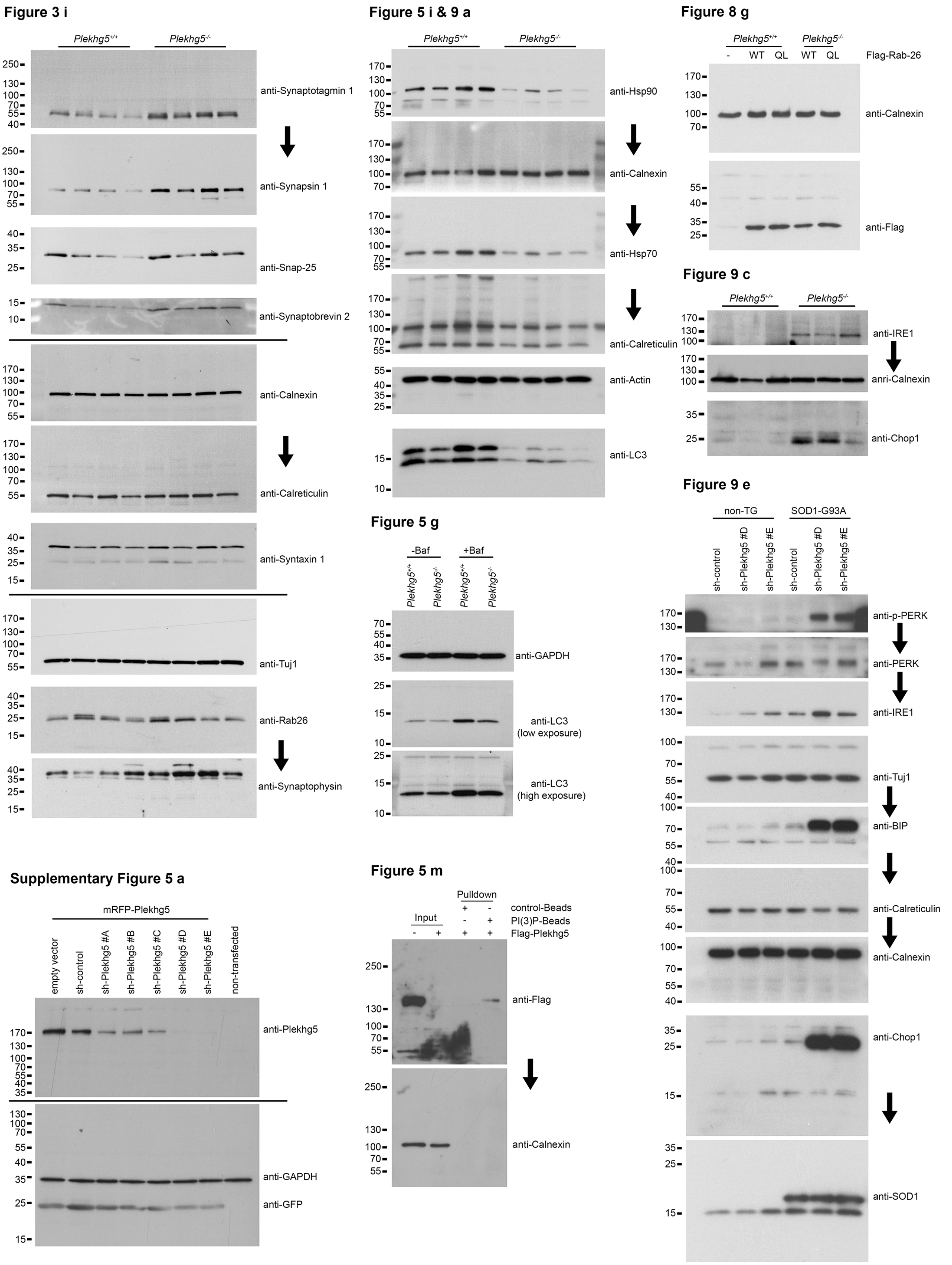

Full size images of Western blots. Arrows indicate reprobing of Western blots. Lines separate individual Western blots.
